# Correspondence

**Published:** 1880-09

**Authors:** A. F. Angell

**Affiliations:** Newport, R. I.


Very respectfully yours,

J. C. Beckham, M. D.

---

### CORRESPONDENCE.

Newport, R. I., August 8th, 1880.

*Dr. B. M. Wilkerson.*

Dear Sir:—I am practicing Medicine as well as Dentistry, and believe I am a better dentist for being a physician, and a better physician for being a dentist, and yet it is doubtful if I am as well rewarded for my labors for practicing both as if I confined my efforts to either exclusively.

From having studied both professions, I am often able to settle questions satisfactorily that would puzzle me in either, if I had not studied both.

In 1870 after treating several nervous patients with Belladonna, and hearing them complain of the dryness of their mouth, and recollecting that Headland gave the dryness of the throat as one of the peculiar characteristics of Belladonna. I thought this medicine could be utilized in the practice of dentistry. Not long after, I had several lower molars to fill where it was difficult to use the rubber dam.

To several young persons I gave 5 to 6 drops of the fluid extract of Belladonna when I commenced my operations, for it will take half an hour before its effects are very perceptible in drying the mouth, and it will continue three or four hours. Perhaps in a dose of ten drops, its most decided effects will be felt in from two to three hours after the dose is taken.

After a few successful trials, a young lady aged 24 had several large difficult cavities to be filled in her lower teeth. I gave her five drops of the fluid extract of Belladonna and commenced operations which continued for four hours, with no trouble from the saliva.

When the operation was completed, the lady expressed great satisfaction, from the effects of the medicine in quieting her nervous sensations. I have often used Belladonna since with great satisfaction, and with no bad effects. I seldom find it necessary to give more than ten drops. For a child in filling temporary teeth from two to five drops have often given great satisfaction both in its effects on the nerves and saliva.

If there are any ideas in the foregoing statements that you deem fit for communication, to the profession, they are at your service. If too lengthy boil them down as much as you please.

Very respectfully yours,

A. F. Angell, M. D.

